# Impact of interstitial lung disease and simultaneous lung cancer on therapeutic possibilities and survival

**DOI:** 10.1111/1759-7714.13481

**Published:** 2020-05-13

**Authors:** Eniko Barczi, Tamas Nagy, Livia Starobinski, Abigel Kolonics‐Farkas, Noemi Eszes, Aniko Bohacs, Adam Domonkos Tarnoki, David Laszlo Tarnoki, Veronika Müller

**Affiliations:** ^1^ Department of Pulmonology Semmelweis University Budapest Hungary; ^2^ Medical Imaging Centre Semmelweis University Budapest Hungary

**Keywords:** Interstitial lung disease, lung cancer, nintedanib, survival, therapy

## Abstract

**Background:**

Fibrosing interstitial lung diseases (ILDs) are associated with poor survival and an increased risk of developing lung cancer (LC). Patient and LC characteristics, therapeutic possibilities and survival in this rare patient population are not well established.

**Methods:**

Fibrosing ILD patients treated at the Department of Pulmonology Semmelweis University were reviewed retrospectively between 2012–2018 (*N* = 160). All patients with concomitant LC (*N* = 23) underwent detailed pulmonary evaluation. Cancer characteristics including driver mutation data, as well as therapy and survival were analyzed.

**Results:**

ILD‐LC patients (56% men, mean age 73 ± 6 years) had mild‐moderate lung functional impairment (forced vital capacity [FVC]: 80 ± 24%ref., forced expiratory volume in one second [FEV1]: 76 ± 27%ref.; transfer factor of the lung for carbon monoxide [TLCO]: 62 ± 25% reference). In 56% of cases histology confirmed adenocarcinoma followed by squamous cell carcinoma in 26%. Lobectomy could only be performed in one case; driver mutation was present in one patient. Chemotherapy was most commonly administered; however, 26% could only receive supportive palliative care. Four idiopathic pulmonary fibrosis patients received concomitant nintedanib to their LC treatment. Median survival of ILD‐LC patients was only 321 days.

**Conclusions:**

Diagnosis and therapy of ILD‐LC is challenging and patients have a very limited survival. A significant proportion of patients could only receive palliative care indicating the need for better management strategies in this special patient population. The evaluation of the effect of cotreatment with antifibrotics needs further study.

**Key points:**

- Interstitial lung diseases are often associated with lung cancer
- Diagnosis is challenging and therapy often limited due to underlying lung disease. Patients received platinum based chemotherapy or only supportive care.

## Introduction

Interstitial lung diseases (ILDs) are often irreversible and progressive with poor prognosis.^1^ Idiopathic pulmonary fibrosis (IPF) is one of the most common ILD with the highest mortality.^2^ Actually available antifibrotic therapies are decreasing lung function loss; however, there are no drugs available to repair damaged lung tissue or reverse progression.^3^, ^4^

Lung cancer (LC) is the leading cause of malignancy‐associated mortality in Hungary.^5^ LC prevalence in patients with IPF varies from 4% to 48%, making it the most serious comorbidity.^6^ Pulmonary fibrosis is a proven risk factor for LC development, as relationship between fibrotic areas and lung carcinogenesis, referred to as “scarcinoma” is described.^7^ Advanced age, male sex, smoking history and simultaneous emphysema are among the main risk factors for lung carcinogenesis in IPF patients.^8^

In ILDs, especially in advanced fibrosing lung disease, histological verification is often challenging due to impaired lung function, comorbidities and higher risk of complications. Delay in diagnosis and less access to adequate tissue samples contributes to limited treatment options. Concomitant lung diseases (eg, chronic obstructive lung disease, emphysema, ILDs, lung infections) are often delaying or complicating diagnosis and/or treatment.^9^

As LC is often associated with reduced survival and ILDs are frequently progressive with shorter life expectancy, our aim was to analyze LC patients in our Hungarian ILD cohort. Therapeutic possibilities and outcome were analyzed in both sexes.

## Methods

### Study population

ILD patients from the Department of Pulmonology Semmelweis University were retrospectively reviewed during the period of 1 November 2012 to 1 November 2018. Systematic search revealed 160 patients with ILD diagnosis. Out of these patients, 23 were diagnosed with simultaneous LC. LCs were classified according to the World Health Organization classification. Staging of LC has been established by the actual TNM seventh and eighth editions accordingly.^10^, ^11^

ILD was always classified at the multidisciplinary team discussion which included a pulmonologist, medical oncologist, radiologist and immunologist, based on American Thoracic Society (ATS)/European Respiratory Society (ERS)/Japanese Respiratory Society (JRS)/ Latin American Thoracic Association (ALAT) official guidelines.^12^, ^13^ ILD consisted of IPF (*N* = 18), connective tissue disease (CTD)‐ILD (*N* = 3) and nonspecific interstitial pneumonia (NSIP) (*N* = 2). All IPF patients were also enrolled into the European MultiPartner IPF Registry (EMPIRE).^14^, ^15^

### Pulmonary evaluation

At baseline and every follow‐up physical examination was performed, and a detailed medical history was taken with special emphasis on symptoms (dry/productive cough, sputum, chest pain), respiratory infections and comorbidities. Lung function measurements included functional vital capacity (FVC), forced expiratory volume in 1 second (FEV1), FEV1/FVC and total lung capacity (TLC) by means of electric spirometer and plethysmography (PDD‐301/s, Piston, Budapest, Hungary) according to the American Thoracic Society and European Respiratory Society guidelines.^16^ The highest of three technically acceptable maneuvers was used. Transfer factor of the lung for carbon monoxide (TLCO) was measured with single breath CO method (PDD‐301/s, Piston, Budapest, Hungary) and coefficient (KLCO) calculated. Lung function variables were expressed as percentage of predicted values. Arterialized capillary blood gases, pH and bicarbonate levels were analyzed at rest at room temperature (Cobas b 221, Roche, Budapest, Hungary). Gender‐age‐physiology (GAP) score was calculated for all ILD patients.^17^

### Lung cancer assessment

High resolution computed tomography (HRCT) examination was performed. LC cell type, epidermal growth factor (EGFR), KRAS and if available programmed death ligand‐1 (PD‐L1) data were collected, as well as Eastern Cooperative Oncology Group Performance Status (ECOG PS). Therapy modalities were summarized, and treatment outcomes with special emphasis on survival.

### Ethical statement

Written informed consent was obtained from all subjects prior to their admission to the EMPIRE registry (TUKEB 69/2015). As this was a retrospective real‐world analysis no registration as a clinical trial was needed.

### Statistical analysis

Statistical analysis was performed using Graph Pad software (GraphPad Prism 5.0 Software, Inc., La Jolla, CA, USA). Data are expressed as mean ± standard deviation. Differences between groups for parametric data were evaluated with Student's *t*‐test after testing for normality using a Kolmogorov‐Smirnov test, while chi‐square test was applied for analyzing categorical data. Examinations could not be performed in all cases due to the health state of patients, and the actual number of analyzed patients is reported in the respective tables. Survival was estimated using the Kaplan‐Meier method and calculated from the diagnosis of LC. A *P*‐value <0.05 was defined as statistically significant.

## Results

From the 160 ILD patients analyzed 14% were identified with concomitant LC. Patient characteristics are summarized in Table 1. Patients were older age, and men were significantly older than women. GAP score, and a widely used mortality risk assessment tool for IPF, showed that 32% of the patients were in stage II, while 27% were in stage III. Comparing the two genders the difference was significant, more women being in GAP stage I. Majority of patients had two or three comorbidities, the two most frequent conditions were hypertension and type 2 diabetes.

**Table 1:** Patient characteristics

Parameters	All patients	Women	Men	*P*‐value
	*N* = 23	*N* = 10	*N* = 13	(women vs. men)
Age (years)	73.8 ± 6.2	70.40 ± 6.15	76.54 ± 5.04	**0.02**
Smoking: N (%)				
Former smoker	19 (83)	7 (70)	12 (92)	0.28
Never smoker	4 (17)	3 (30)	1 (8)	0.16
BMI (kg/m2)	25.33 ± 5.17	25.73 ± 6.17	25.03 ± 4.55	0.76
ECOG PS: N (%)				
0–1	7 (30)	3 (30)	4 (31)	0.96
2	14 (61)	6 (60)	8 (61)	0.94
3–4	2 (9)	1 (10)	1 (8)	0.84
GAP: N (%)				
Stage I	10 (43)	7 (70)	3 (23)	**0.02**
Stage II	7 (32)	3 (30)	4 (33)	0.99
Stage III	6 (27)	0	6 (50)	**0.05**
Comorbidities: N (%)				
0	2 (9)	1 (10)	1 (8)	0.99
1	2 (9)	1 (10)	1 (8)	0.99
2	12 (52)	5 (50)	7 (53)	0.99
3	7 (30)	3 (30)	4 (31)	0.99
Symptoms: N (%)				
Dyspnea	17 (74)	8 (80)	9 (69)	0.55
Cough and sputum	16 (70)	7 (70)	9 (69)	0.96
Chest pain	4 (17)	2 (20)	2 (15)	0.77
HRCT: N (%)				
pUIP	15 (65)	6 (60)	9 (69)	0.64
UIP	8 (35)	4 (40)	4 (31)	0.64
ILD disease: N (%)				
CTD‐ILD or NSIP	5 (22)	3 (30)	2 (15)	0.39
IPF	18 (78)	7 (70)	11 (85)	0.39

BMI, body mass index; CTD, connective tissue disease; ECOG PS, Eastern Cooperative Oncology Group Performance Status; GAP, gender‐age‐physiology; HRCT, high resolution CT; ILD, interstitial lung disease; IPF, idiopathic pulmonary fibrosis; NSIP, nonspecific interstitial pneumonia.

Functional parameters are summarized in Table 2. Lung function showed a mild restrictive ventilatory pattern, without differences between genders in predicted values. Blood gases showed higher pCO_2_ in women as compared to men, without differences in pO_2_.

**Table 2:** Lung function and capillary blood gas values at baseline

Parameters	All patients	Women	Men	*P*‐value
	*N* = 23	*N* = 10	*N* = 13	(women vs. men)
Lung function and diffusion test				
FVC (L)	2.48 ± 0.82	1.94 ± 0.70	2.93 ± 0.75	**0.01**
FVC (%)	80.80 ± 24.00	78.70 ± 28.20	82.60 ± 20.90	0.71
FEV1 (L)	1.81 ± 0.70	1.42 ± 0.58	2.15 ± 0.63	**0.01**
FEV1 (%)	75.50 ± 26.70	70.90 ± 32.60	79.30 ± 21.40	0.47
FEV1/FVC	0.90 ± 0.20	0.90 ± 0.20	1.00 ± 0.20	0.29
TLC (L)	4.09 ± 1.36	3.90 ± 1.70	4.34 ± 0.80	0.51
TLC (%)	75.80 ± 26.50	77.50 ± 30.20	74.10 ± 23.90	0.77
TLCO (mmol/min/kPa)	4.47 ± 2.11	3.95 ± 2.04	5.32 ± 2.15	0.27
TLCO (%)	61.80 ± 24.90	60.20 ± 28.30	64.20 ± 21.00	0.77
Capillary blood gas test				
pH	7.42 ± 0.03	7.40 ± 0.03	7.43 ± 0.02	**0.05**
pCO_2_ (mmHg)	36.96 ± 4.58	40.6 ± 4.16	34.78 ± 3.35	**0.01**
pO_2_ (mmHg)	61.62 ± 8.60	57.17 ± 7.43	64.29 ± 8.46	0.11

TLCO, transfer factor of the lung for carbon monoxide; FVC, forced vital capacity; FEV1, forced expiratory volume in 1.0 seconds; TLC, total lung capacity

Histology verified adenocarcinoma in 13 patients (56%) being the most LC type in this cohort, while six patients (26%) had squamous cell lung cancer. Small cell lung cancer (SCLC) was found only in two patients and in two cases only non‐small cell lung cancer (NSCLC) diagnosis could be established (Table 3). Cause of death in the majority of patients was progression of lung cancer (86%), while advanced lung fibrosis was the cause in 14% of cases.

**Table 3:** Lung cancer histology, stage and mutation type, cancer treatment and cause of death

Parameters	All	Women	Men	*P*‐value
	*N* = 23	*N* = 10	*N* = 13	
Histology: N (%)				
Adenocarcinoma	13 (56)	7 (70)	6 (46)	0.25
Squamous cell lung cancer	6 (26)	1 (10)	5 (39)	0.12
Small cell lung cancer (SCLC)	2 (9)	1 (10)	1 (8)	0.85
Other (non‐small cell lung cancer [NSCLC])	2 (9)	1 (10)	1 (8)	0.85
TNM: N (%)				
Local (I, II, IIIA)	9 (39)	5 (50)	4 (31)	0.34
Locally advanced/metastatic (IIIB, IV)	14 (61)	5 (50)	9 (69)	0.34
Adenocarcinoma Mutation type: N (%)				
*KRAS* mutant	4 (31)			
*EGFR* mutant	1 (7)			
*EGFR*, *KRAS* wild‐type	8 (62)			
Treatment: N (%)				
Lobectomy	1 (4)			
Platinum doublet therapy +/− irradiation	12 (52)			
Mono chemotherapy +/− irradiation or only irradiation	4 (17)			
Best supportive care				
Not receiving active oncotherapy	5 (22)			
Refusing active oncotherapy	1 (4)			
Cause of death: N (%)				
Progressive lung fibrosis	3 (14)			
Progression of lung cancer	18 (86)			

At the time of the LC diagnosis 14 patients (61%) had locally advanced or metastatic stage (IIIB, IV) disease. Early local stages (I, II, IIIA) were diagnosed in 39% of all patients. Among patients diagnosed with adenocarcinoma, mutational analysis was performed in 13 cases, one patient showed *EGFR* mutation, four patients *KRAS* mutation, while PD‐L1 expression was confirmed in three samples. More men had squamous cell lung cancer as compared to women.

PS was mainly two (61%), and only 30% of patients were fit enough (PS 0–1) for multiple treatment modalities.

LC was treated according to the histology type, TNM, PS and treatment availability at the time of diagnosis. Each case was discussed by the onco‐team. Treatments are summarized in Table 3. Lobectomy could only be performed in one case. Chemotherapy was most commonly administered; however, six patients (26%) could only receive best supportive palliative care. Four IPF patients received nintedanib in addition to their concomitant LC treatment.

**Figure 1:**
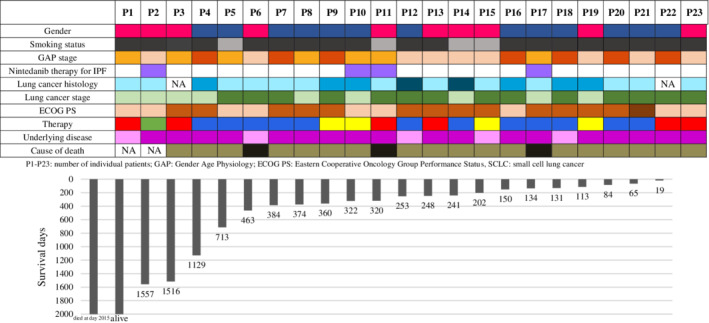
Summary of patients, disease characteristics and therapies of individual patients. (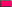) Female; (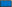) Male; (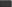) Former smoker; (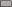) Never smoker; (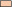) GAP I; (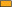) GAP II; (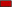) GAP III; (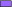) Nintedanib therapy; (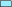) Adenocarcinoma; (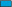) Squamous cell carcinoma; (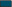) Small cell lung cancer; (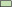) Early stage (I, II, IIIA); (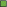) Locally advanced/metastatic (IIIB, IV); (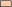) ECOG 0–1; (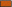) ECOG 2; (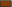) ECOG 2–3; (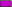) IPF; (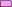) CTD‐ILD or NSIP; (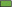) Lobectomy; (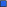) Platinum doublet therapy +/‐ irradiation; (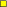) Mono chemotherapy +/‐ irradiation or only irradiation; (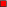) Not receiving/refusing active oncotherapy; (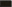)Progressive lung fibrosis; (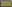) Progression of lung cancer.

Treatment outcomes are individually shown in Fig 1. One patient with the longest survival, who was still alive in 2019, had a small tumor, underwent lobectomy and still receives nintedanib.

**Figure 2:**
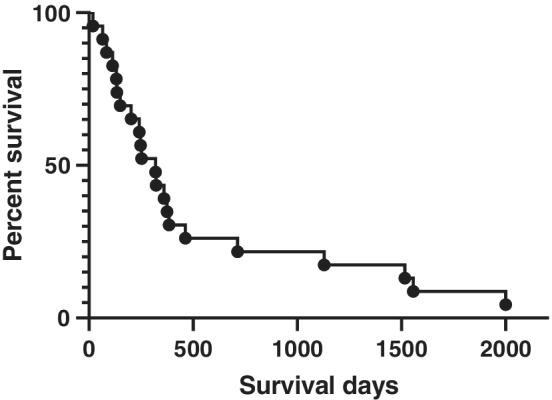
Survival curve of ILD‐LC patients. The average survival was 321 days from the diagnosis of LC (men: 340 days, women: 288 days; ns) in ILD patients.

Median survival was 321 days, lower as compared to the most severe IPF (FVC < 60%; *N* = 22; average median survival 460 days) population as published previously.^18^ Median survival among in our cohort for males was 340 days, while in women 288 days. No difference between sexes was noted in median survival (*P* = 0.643) (Fig 2).

## Discussion

We confirmed 23 LC cases in ILD patients. In a previous study, 2% of the non‐IPF‐ILD and 3% of IPF patients developed simultaneous LC,^19^ and this rate was higher in our cohort (7% and 6%). The first studies of ILD associated LC appeared in the last decades and several reviews discuss the mechanism of LC development in ILDs.^20^, ^21^

In our study, 15 patients were diagnosed with ILD first and cancer developed after a median of 888 days (range 19–1557) which is in line with previous observations as LC is mostly diagnosed after ILD.^22^ When a working diagnosis of IPF is made, the patient should be closely monitored, and clinical, radiological, and laboratory assessments repeated as appropriate so that their diagnosis can be reviewed at regular intervals (ideally based on further rounds of MDD).^23^, ^24^ In another eight cases, cancer and ILD were diagnosed simultaneously.

LC is often diagnosed late in Hungary, leading to high mortality,^5^ and adenocarcinoma is the most frequent LC type. Similarly, in our cohort, adenocarcinoma was the most frequently diagnosed type of LC (56%), in contrast with other studies which found squamous cell carcinoma more common (41% vs. 26%), ^19^ or a similar distribution was revealed.^20^ The majority of our patients already had metastatic disease, also in line with previous observations, as diagnosis is often late and a significant proportion of the patients did not meet the criteria to receive combined chemotherapy.^9^

Activating mutations in the *EGFR* gene was detected in only one case, and 62% of the patients showed *EGFR*/*KRAS* wild phenotype while PD‐L1 > 1% expression was found in three cases. In Hungary, *KRAS* testing is routinely performed due to special reimbursement issues. Previous data confirmed 32.1% *KRAS* mutation lung adenocarcinoma in our country, which is within the average (25%–35%) of other observations.^25^, ^26^ In our ILD patients with lung adenocarcinoma four patients (31%) were harboring the *KRAS* mutation, three had UIP and one CTD‐ILD. This is in line with a previous study where less frequent *EGFR* and more frequent *KRAS* mutations were observed in UIP‐positive cases.^27^

Operability was rarely applied in our study, and only one patient was suitable for lobectomy. Platinum doublet therapy +/− irradiation was most frequently used (52%), although radiotherapy was applied carefully because of the possibility of radiation pneumonitis in severe ILD.^28^ Recent clinical trials assessing the effect of immunotherapy alone, or in combination with chemotherapy, excluded patients with ILDs as it might increase severe immune‐related pneumonitis.^29^, ^30^

In a significant proportion of patients, PS did not allow detailed examination and/or more effective oncotherapy due to their underlying lung disease. High tumor burden and poor PS often limit therapeutic possibilities. Five patients received only best supportive care and one patient refused all suggested therapies.

In four out of 11 patients with IPF, additional therapy with nintedanib was used. Nintedanib is widely used for the treatment of IPF as it is slows progression of functional decline.^31^ It is important to note that nintedanib has only been available in Hungary for IPF treatment since 2015, and is the main reason why only 36% of patients received this drug. The efficacy of nintedanib in advanced NSCLC in combination with chemotherapy has been reported in the LUME‐Lung 1 and LUME‐Lung 2 trials.^32^ Nintedanib may play a role in the treatment of IPF‐associated lung cancer as it can slow progression of both diseases individually^31^, ^33^, ^34^ however, no data on IPF and LC treatment are available.

Pirfenidone, the other antifibrotic therapy used in the treatment of IPF, has shown advantages for decreased LC development rates.^35^ In our cohort, none of our patients received pirfenidone, as this drug was reimbursed later as nintedanib by the Hungarian insurance system.

In conclusion, median survival was 321 days from the diagnosis of LC (men: 340 days, women: 288 days) in ILD patients. The prognosis of patients with ILD‐LC is similar to that for patients with advanced IPF only.

Therapeutic options are limited: operability was only possible in one early stage tumor and 26% of the patients were not fit enough for chemotherapy due to PS ≥2 and/or underlying lung disease, or comorbidities. Interdisciplinary evaluation of therapeutic options is mandatory for the final decision in concomitant ILD‐LC therapy. Further investigations and larger patient group are needed to evaluate the possible protective effect of antifibrotic drugs in ILD patients for LC development.

## Disclosure

All authors declare that they have no conflict of interests.
